# A non-invasive iRFP713 p53 reporter reveals dynamic p53 activity in response to irradiation and liver regeneration in vivo^†^

**DOI:** 10.1126/scisignal.abd9099

**Published:** 2022-02-08

**Authors:** Timothy J Humpton, Andreas K Hock, Christos Kiourtis, Marco De Donatis, Frederic Fercoq, Colin Nixon, Sheila Bryson, Douglas Strathdee, Leo M. Carlin, Thomas G. Bird, Karen Blyth, Karen H Vousden

**Affiliations:** 1 The Francis Crick Institute, London, NW1 1AT, United Kingdom; 2 Cancer Research UK Beatson Institute, Glasgow, G61 1BD, United Kingdom; 3 Institute of Cancer Sciences, University of Glasgow, Glasgow, G61 1QH, United Kingdom; 4 Institute of Cancer Sciences, University of Glasgow, Glasgow, G61 1QH, United Kingdom; 5 MRC Centre for Inflammation Research, The Queen’s Medical Research Institute, University of Edinburgh, EH164TJ, United Kingdom

## Abstract

Genetically encoded probes are widely used to visualize cellular processes in vitro and in vivo. Although effective in cultured cells, fluorescent protein tags and reporters are sub-optimal in vivo due to poor tissue penetration and high background signal. Luciferase reporters offer improved signal-to-noise ratios but require injections of luciferin that can lead to variable responses and that limit the number and timing of data points that can be gathered. Such issues in studying the critical transcription factor p53 have limited insight on its activity in vivo during development and tissue injury responses. Here, by linking the expression of the near-infrared fluorescent protein iRFP713 to a synthetic p53-responsive promoter, we generated a knock-in reporter mouse that enabled non-invasive, longitudinal analysis of p53 activity in vivo in response to various stimuli. In the developing embryo, this model revealed the timing and localization of p53 activation. In adult mice, the model monitored p53 activation in response to irradiation and paracetamol-or CCl_4_- induced liver regeneration. After irradiation, we observed potent and sustained activation of p53 in the liver, which limited the production of reactive oxygen species (ROS) and promoted DNA damage resolution. We propose that this new reporter may be used to further advance our understanding of various physiological and pathophysiological p53 responses.

## Introduction

The p53 tumor suppressor protein is a transcription factor that is induced in response to stress to mediate various outcomes ranging from cell survival to cell death ^1–3^. In vitro studies have shown that a wide range of signals can activate p53, resulting in induction of the expression of a large number of p53 target genes ^2^. Although many studies have documented the various transcriptional programs induced by p53, the p53 response over time to various stress signals in vivo are less well understood. To address these questions, several transgenic mouse models expressing p53-reporter genes have been described. Mice carrying a lacZ p53 reporter has demonstrated p53 activity in embryos and a strong induction of p53 in response to irradiation (IR) in embryonic and radiosensitive adult tissues ^4,5^, but the approach required tissue fixation for ß-galactosidase detection. Non-invasive imaging of a mouse carrying a p53-reponsive luciferase reporter showed induction of p53 activity in live mice in response to IR, with evidence of oscillations of p53 activity over time ^6^. Unfortunately, epigenetic silencing of the reporter transgene limited the utility of this model ^7^. In further studies, p53-reponsive elements from the *Cdkn1a/p21* or *Bbc3/Puma* promoters were used to make enhanced green fluorescent protein (EGFP) reporter genes, which were integrated into the *Hprt* locus to avoid silencing. These mice confirmed p53 activity in early embryos ^8^, but detection of EGFP in live mice was not reported, possibly because probes that fluoresce in the visible light spectrum—including GFP and red fluorescent protein (RFP)—are limited by poor tissue penetration and high autofluorescence ^9,10^. Although luciferase overcomes some of these limitations, it requires the injection and subsequent diffusion of exogenous luciferin to sites of luciferase activity in order to function. Because the bioavailability of luciferin can vary widely between well-perfused and poorly vascularized tissues, which include many tumors, absolute quantification of luciferase data can be difficult ^11–13^.

Proteins that fluoresce in the near infrared (NIR) spectrum have several advantages over commonly used probes, such as GFP and luciferase ^14^. We have previously shown that one such NIR protein, iRFP713 (hereafter referred to as iRFP), can be readily detected in vivo and have successfully used an endogenous inducible knock-in iRFP mouse (LSL-iRFP) to non-invasively monitor tumor growth in genetic models of cancer ^15^. Given the deep tissue penetration, rapid image acquisition time, and superior signal-to-noise characteristics of this platform ^15–17^, we sought to expand the utility of iRFP-based imaging by using iRFP to track p53 transcriptional activity in vivo.

To this end, we have generated p53 iRFP reporters that allow for rapid and non-invasive longitudinal visualisation of the p53 response. Our work introduces a new tool to study p53 activity both in vitro and in vivo, thereby opening up avenues for research into the timing and tissue localization of both canonical and emerging non-cancer roles of p53 activity in the mouse.

## Results

### P53rep-iRFP reporter recapitulates dynamics of p53 activation in vitro

**Figure 1:**
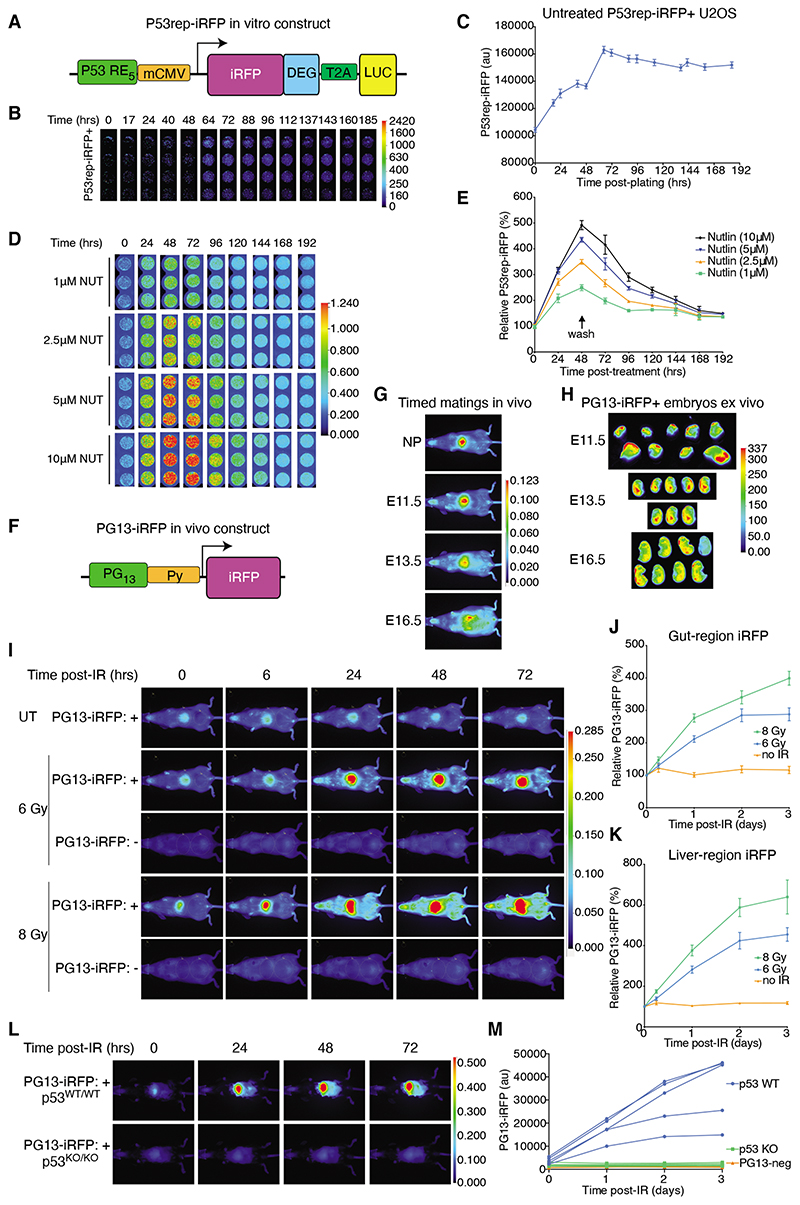
P53-iRFP reporters recapitulate the dynamics of p53 activity in vitro and in vivo. **(A)** Schematic of the P53rep-iRFP in vitro construct. DEG: destabilisation domain. LUC: Luciferase. mCMV: minimal CMV promoter p53 RE_5_: 5-repeat consensus p53 binding sequences. Further details in the Methods. **(B and C)** Time-series images (B; hours post-plating) and quantification (C) of iRFP fluorescence in stable P53rep-iRFP+ U2OS cells over time. Lookup table (LUT) profile used for all images shown. Images are from four technical replicates, and quantification of N=2 independent sets (8 replicates in total) are shown, mean ± SD. **(D and E)** Time-series (D, hours post-treatment) and quantification (E) of iRFP relative to matched untreated baseline iRFP level in stable P53rep-iRFP+ U2OS cells treated with Nutlin (NUT) as indicated, with its removal at 48 hours (“wash”, E). Representative images are from technical replicates of one experiment and quantification is from three independent experiments; shown as mean ± SEM. **(F)** Schematic of the PG13-iRFP in vivo construct. Expression of iRFP is driven by a Py: polyoma virus early gene promoter. PG_13_: 13-repeat consensus p53 binding sequences. Further details are provided in the Methods. **(G)** PG13-iRFP in vivo signal intensity within a non-pregnant (NP) PG13-iRFP+ female mouse and in PG13-iRFP+ female mice at days E11.5, E13.5, and E16.5 of pregnancy (day of plug counted as E0.5). Images are representative of N=2 mice per time point. **(H)** Images of ex vivo iRFP signal (AU) from E11.5, E13.5, and E16.5 embryos arising from timed matings between PG13-iRFP^+/+^ female and PG13-iRFP WT male mice (E16.5) or from PG13-iRFP^+/+^ female and PG13-iRFP^+/y^ male mice. Representative of N=17 E11.5 embryos, N=8 E13.5 embryos, and N=17 E16.5 embryos from N=2 timed matings per time point. The corresponding pregnant female for embryos arising from one of these timed matings per time point is shown in (H). **(I)** Representative images of PG13-iRFP+ and control (PG13-iRFP-negative) mice treated with either 6 Gy or 8 Gy TBI and analysed 72 hours after treatment as indicated. N=6 mice per irradiated group at 0 hours, 3 at 6 hours, 6 at 24 hours, 5 at 48 hours, and 4 at 72 hours; N=5 untreated (UT) mice. Note: UT and 6 Gy PG13-iRFP+ mice here are the same as depicted longitudinally in fig. S2H; therefore, the time 0 images there are the same. **(J and K)** Quantification of gut-region (J) and liver-region (K) iRFP intensity relative to baseline (time-point 0) of PG13-iRFP+ mice described in (I). Data are mean ± SEM. N as detailed in (I); time, in hours. **(L)** Representative images of PG13-iRFP+; *p53^WT/WT^* and PG13-iRFP+; *p53^KO/KO^* mice treated with 8 Gy TBI and imaged daily for 3 days thereafter. N=5 mice per genotype. This colony had black coat color, and all mice were depilated prior to baseline imaging. **(M)** Quantification of liver-region iRFP (au) in the mice described in (L), alongside same-condition results from N=3 PG13-iRFP-negative mice. Lines each correspond to an individual mouse.

Based on previous experience generating an inducible lox-STOP-lox (LSL)-iRFP construct that allows for the monitoring of cell proliferation both in vitro and in vivo^15,18^, we created an iRFP-based p53 reporter system, P53rep-iRFP, to monitor p53 transcriptional activity in vitro (Fig. 1A). In this system, p53 binding to consensus response elements drives the expression of iRFP (Fig. 1A)^19–21^. A destabilization domain was added to the C-terminus of iRFP in the P53rep-iRFP construct to promote its rapid degradation (Fig. 1A)^22^, allowing for an assessment of the rate of recovered control over p53 activity following the removal of a p53-activating signal. This P53rep-iRFP construct is comparable to the PG13-luciferase reporter ^23^—albeit with fewer p53 response elements and a destabilized reporter. Like the PG13-luciferase reporter, our initial in vitro reporter expressed Luciferase as well as iRFP, but this functionality was ultimately not required and was not retained in the subsequent in vivo targeting construct.

During normal cell growth, the expression of iRFP in U2OS cells that stably expressed the P53rep-iRFP plasmid was low (Fig. 1, B and C), with a mild increase in iRFP fluorescence over the first 72 hours after plating (Fig. 1, B and C), consistent with an increasing cell density and then confluence ^24^. Treatment of the U2OS cells with Nutlin-3A (hereafter, Nutlin), a potent p53-activating compound that inhibits the MDM2-p53 interaction and causes rapid stabilisation of p53 ^25^, increased P53rep-driven iRFP signal in a dose-dependent manner (Fig. 1, D and E). Removal of Nutlin after 48 hours led to iRFP signal decay back to baseline within four days, reflecting a combination of the in vitro half-life of Nutlin, the speed of restored control over p53 signalling, and the rate of degradation of the residual iRFP. These findings are consistent with published kinetics for the p53 response to Nutlin and suggest that iRFP fluorescence from the P53rep-iRFP construct accurately reflects both the timing and relative intensity of p53 activity in vitro ^26^.

The p53 pathway is activated in response to a variety of chemotherapeutic agents, including actinomycin D (ActD), 5-fluorouracil (5-FU), adriamycin (doxorubicin), etoposide, and cisplatin (fig. S1, A to J) ^27^. Using P53rep-iRFP-expressing cells we confirmed robust activation of p53 in response to two concentrations of these agents. Notably, our system detected two clear classes of kinetic responses. Similar to Nutlin, treatment with low-dose ActD (which induces ribosomal stress but not genotoxic damage) led to a rapid increase in iRFP signal that was parabolic in nature, decreasing to baseline monotonically after removal of the drug (fig. S1, A and B). In contrast, the iRFP signal following treatment with adriamycin, 5-FU, and cisplatin plateaued and decreased more slowly after washout of the treatment (fig. S1, C to J). These observations suggest that a p53-activating signal persists after removal of these DNA-damaging drugs and is reminiscent of the bimodal modulation of p53 activity after chemotherapy treatment observed at the single-cell level in a previous report^28^.

### PG13-iRFP reporter mouse reveals activation of p53 in the developing embryo and after irradiation in adult mice

To explore the potential of a p53 iRFP reporter in vivo, we generated a knock-in transgenic mouse strain that expresses iRFP in response to p53 activation (Fig. 1F and fig. S2A). To allow for a stronger signal that would be more likely to be detected by non-invasive imaging of the whole mouse, we omitted the destabilization domain, thus maximising the potential for signal accumulation over time. One benefit of this system is that the resulting stability of iRFP signal effectively marks tissues where p53 has been engaged, providing a longer period of time to identify these compartments after transient activation of p53. As a trade-off, however, removal of the destabilization domain obviated the possibility of observing rapid oscillations in p53 activity such as those reported in the response to irradiation^6^. To increase the signal further, we also expanded the number of p53 binding sequences in our in vivo targeting construct, using thirteen repeats of the consensus p53 binding sequence (PG sequence) that is widely used to monitor p53 activity with the PG13-luciferase reporter in vitro ^20,23,29^. Introducing the PG13-iRFP construct into the endogenous *Hprt* locus on the murine X-chromosome created the *Hprt^Tm1(p53RE-iRFP [pg13])Bea^* mouse strain (the PG13-iRFP mouse) (fig. S2A). This strain was backcrossed onto the Albino C57BL/6J background to allow for unimpeded scanning of PG13-iRFP mice without depilation.

Mice carrying the PG13-iRFP allele were viable, born at expected Mendelian ratios from interbreeding of hemizygous male (PG13^+/y^) and heterozygous female (PG13^+/-^) mice, and indistinguishable from PG13-iRFP wild-type littermates (fig. S2B). Untreated adult PG13-iRFP+ mice showed low levels of baseline iRFP expression, albeit with a consistently low but detectable signal from the liver/spleen-region that was stable over time (fig. S2, C and D). We also observed modest differences in baseline iRFP levels between unstressed PG13-iRFP mice, a variation that appeared greater in female mice (fig. S2C).

To test our in vivo reporter system, we first examined pregnant PG13-iRFP mice to determine whether it was possible to resolve p53 activity in developing embryos. Compared with female littermate mice that were not pregnant, we observed rising iRFP signal in pregnant mice at embryonic day 11.5 (E11.5), E13.5, and E16.5 (Fig. 1 G). However, we were not able to directly visualise embryos above the signal in the liver-region of the pregnant females in vivo (Fig. 1 G). Nevertheless, ex vivo imaging revealed localised intense PG13-iRFP signal in the brain, spine, and bones of E13.5 and E16.5 embryos, consistent with previous studies showing localised activation of p53 earlier in embryogenesis (E3.5 to E12.5) (Fig. 1 H)^4,5,8^. iRFP signal was also observed with similar localisation in E11.5 embryos, but structures and morphology were more difficult to resolve at this embryonic stage (Fig. 1H). For our timed matings at E16.5, we crossed PG13-iRFP homozygous females with PG13-iRFP-negative (WT) males. As such, resulting embryos were either hemizygous (male) or heterozygous (female) for PG13-iRFP. Quantification of the iRFP signal from each resulting embryo confirmed two distinct populations, with decreased iRFP signal observed in heterozygous embryos (fig. S2E), likely due to mosaicism arising from random X-linked inactivation of the reporter allele in these embryos^30^. Subsequent matings using homozygous female and hemizygous male mice to generate E11.5 and E13.5 embryos—yielding all homozygous or hemizygous embryos—did not share this signal discrepancy (fig. S2E). Based on these observations, we used PG13-iRFP-positive males (hemizygous) and homozygous females as much as possible in our experiments with adult mice to limit potential signal variability. In addition, comparisons of iRFP signal intensity relative to the measured baseline for each individual mouse were found to be the most informative for our comparative analyses.

To explore the activity of the PG13-iRFP reporter in adult mice, we irradiated PG13-iRFP-positive mice with either a sub-lethal (6 Gy) or lethal (8 Gy) dose of total body gamma irradiation (TBI). In our model, we were able to detect a dose-dependent increase in iRFP signal intensity by 6 hours after treatment that persisted for at least 3 days (Fig. 1, I to K, and fig. S2F). This observation is consistent with the established rapid activation of p53 after irradiation in vivo^6,31–35^, as well as the persistence of downstream p53 targets in irradiated tissues within the first 72 hours after TBI^33–35^. We were not able to resolve significantly increased IRFP signal in PG13-iRFP reporter mice earlier than 6 hours (Fig. 1, I to K, and fig. S2, F and G), likely a consequence of the time required to accumulate sufficient iRFP from p53 activity to appreciably increase fluorescence at the whole-body level in our system. Continuous monitoring of mice exposed to 6 Gy TBI showed peak iRFP signal intensity at 7 days after TBI with a gradual return to baseline within 7 weeks (fig. S2H). However, this slow rate of recovery likely reflects the stability of iRFP protein^16^, rather than on-going p53 activation—although this extended model would reflect the persistence of long half-life p53-inducible proteins, such as TIGAR (TP53-induced glycolysis and apoptosis regulator) ^36^.

It has been shown that the overexpression of the p53 family members p63 or p73 can activate the PG13 reporter construct in vitro^37,38^. To test whether the iRFP signal we observed in PG13-iRFP reporter mice after TBI treatment was strictly p53-dependent, we generated *p53* knockout mice that retained the PG13-iRFP reporter (*p53^KO/KO^*; PG13-iRFP+). In this system, we observed neither baseline nor induced iRFP signal in *p53^KO/KO^*; PG13-iRFP+ mice following 8 Gy TBI (Fig. 1, L and M), suggesting that the PG13-iRFP reporter is specific for p53 activation in adult mice in vivo. These findings also suggest that the low-level, liver-region iRFP signal observed in all *p53^WT/WT^*; PG13-iRFP+ reporter mice reflects bona fide low-level p53 activity in this region rather than leakiness in our reporter system. Of note, however, because we did not examine *p53^KO/KO^*; PG13-iRFP+ embryos for iRFP signal, we cannot rule out that some of the iRFP expression during embryogenesis is p53 independent.

### PG13-iRFP reporter mouse identifies potent and sustained activation of p53 in the liver and pancreas after irradiation

Previous studies of isolated tissue from p53 reporter mice have shown an activation of the p53 response in radiosensitive tissues (such as spleen, thymus and intestine), with signal predominantly located to the abdomen, oral cavity and paws ^6^. In PG13-iRFP reporter mice, we unexpectedly observed the largest iRFP signal increase centred on the liver and spleen region at both TBI doses (Fig. 1, I to K). Even accounting for the increased baseline signal present in this area of untreated PG13-iRFP+ mice, the magnitude of increased liver-region iRFP signal was approximately double the induction seen in the gut-region.

**Figure 2:**
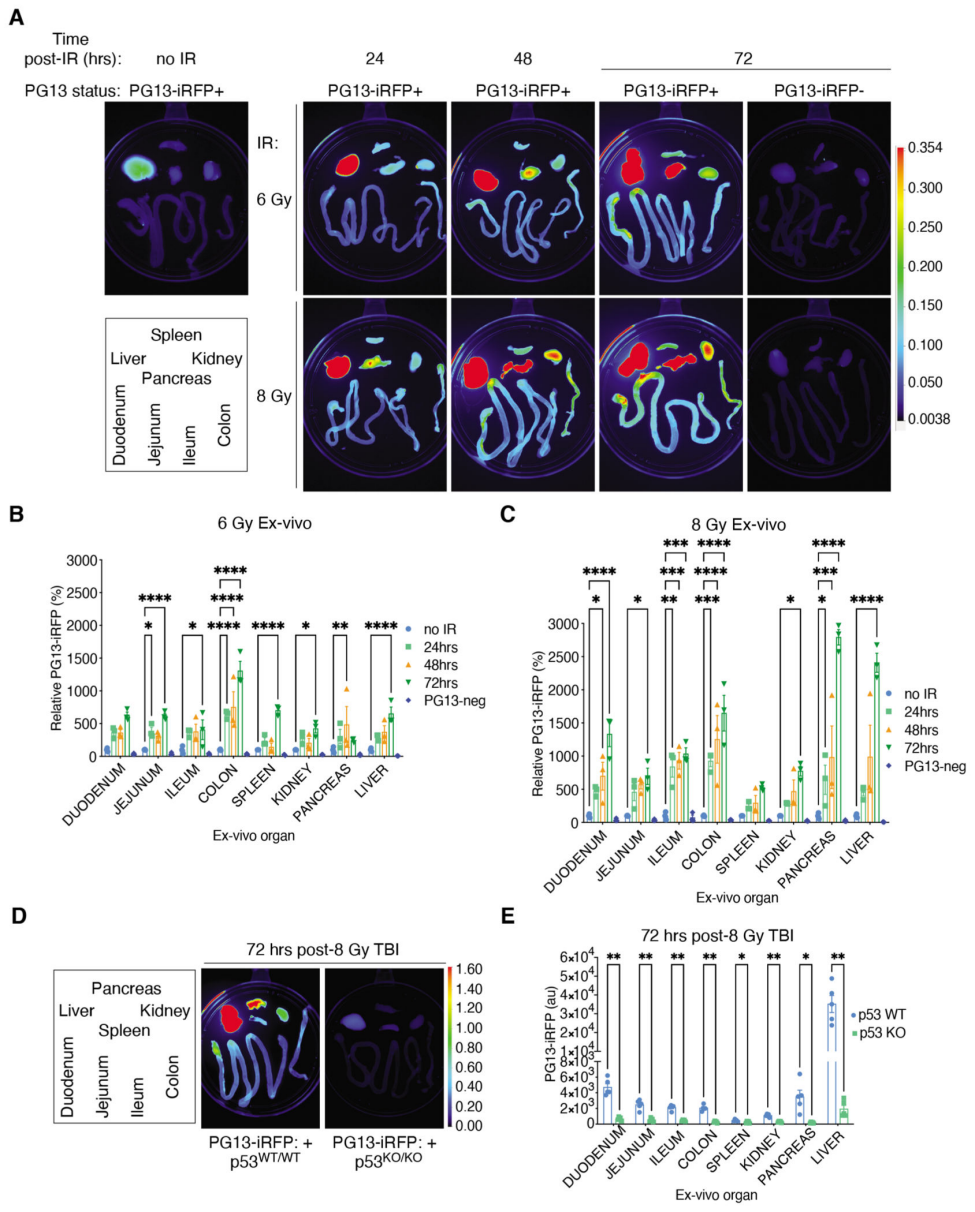
The PG13-iRFP reporter mouse reveals potent and durable p53 activity in the non-radiosensitive pancreas and liver after irradiation. **(A to C)** Representative images (A) and analysis (B and C) of ex vivo tissues from PG13-iRFP+ mice at 0 (no IR), 24, 48, and 72 hours after TBI with 6 Gy or 8 Gy, and of those from control PG13-iRFP- (or “PG13-neg”) mice imaged at 72 hours. LUT profile used for all ex vivo images as shown. Layout of tissues on the plates indicated in the inset legend; not meant to reflect actual positioning within the abdominal cavity. Data are mean ± SEM N=3 mice for each condition. *P<0.05, **P<0.01, ***P<0.001, and ****P<0.0001 by two-way ANOVA with Holm-Sidak’s multiple comparisons test and multiplicity-adjusted *P*-values. **(D and E)** Imaging of ex vivo tissues from PG13-iRFP+; *p53^WT/WT^* and PG13-iRFP+; *p53^KO/KO^* mice treated with 8 Gy TBI and imaged at 72 hours after irradiation. LUT profile and layout as described in (A). Images are representative and data are mean ± SEM of N=5 mice/genotype. *P<0.05 and **P<0.01 by two-way ANOVA with Holm-Sidak’s multiple comparisons test with multiplicity-adjusted *P*-values.

Although it was possible for us to differentiate iRFP signals from regions in two dimensions (such as the liver-region vs. the gut-region) on whole body scans, it was not possible to separate liver-specific signal from that arising from the co-located pancreas, kidney, and spleen. Therefore, for a closer examination of tissue-specific p53 induction, we performed ex vivo analysis of abdominal cavity organs from PG13-iRFP+ mice for 3 days after the 6 Gy and 8 Gy TBI treatments (Fig. 2, A to C). As expected, the PG13-iRFP reporter was rapidly engaged in the spleen, small intestine, and colon, whereas both pancreas and liver also showed robust PG13-iRFP fluorescence in our time-course after both doses of TBI (Fig. 2, A to C). In contrast, we observed a more modest increase in iRFP signal in the kidney. In most tissues, a greater increase in iRFP signal was seen after 8 Gy TBI treatment compared to 6 Gy; the spleen was one exception, most likely reflecting the induction of apoptotic cell death in this organ in response to lethal radiation ^39^. Consistent with our observations from whole body imaging of *p53^KO/KO^*; PG13-iRFP+ mice, organ-specific iRFP signal was also p53-dependent after 8 Gy TBI (Fig. 2, D and E).

Although p53 activation reportedly occurs in the liver at 6 hours after low-dose (5 Gy) TBI ^40^, only very modest p53 activation is detected in the liver of luciferase or lacZ p53 reporter mice in response to IR ^5,6^. To reconcile our observations with these disparate reports, we examined the expression of p21, encoded by a well-validated p53 target gene, in PG13-iRFP mice (fig. S3, A to D). Consistent with both our iRFP data and published reports for p53 signalling after TBI^33–35^, we observed sustained p21 staining in the spleen, small intestine, and colon of PG13-iRFP mice at 3 days after both 6 and 8 Gy TBI treatments. Notably, tissues were histologically similar and exhibited similar induction of p21 in both PG13-iRFP+ and matched PG13-iRFP reporter-negative mice, suggesting that the presence of the p53 reporter did not alter the tissue response to TBI treatment. In both sets of mice, p21 staining correlated with radiation dose. In agreement with our iRFP data and with previous reports ^40^, we also detected increased p21 staining in the liver and pancreas—much more than was observed in the kidney (fig. S3, B to D), confirming that downstream targets of the p53 pathway remained engaged in these organs 3 days after irradiation.

### Induction of liver p53 promotes DNA repair and ROS control after irradiation

**Figure 3:**
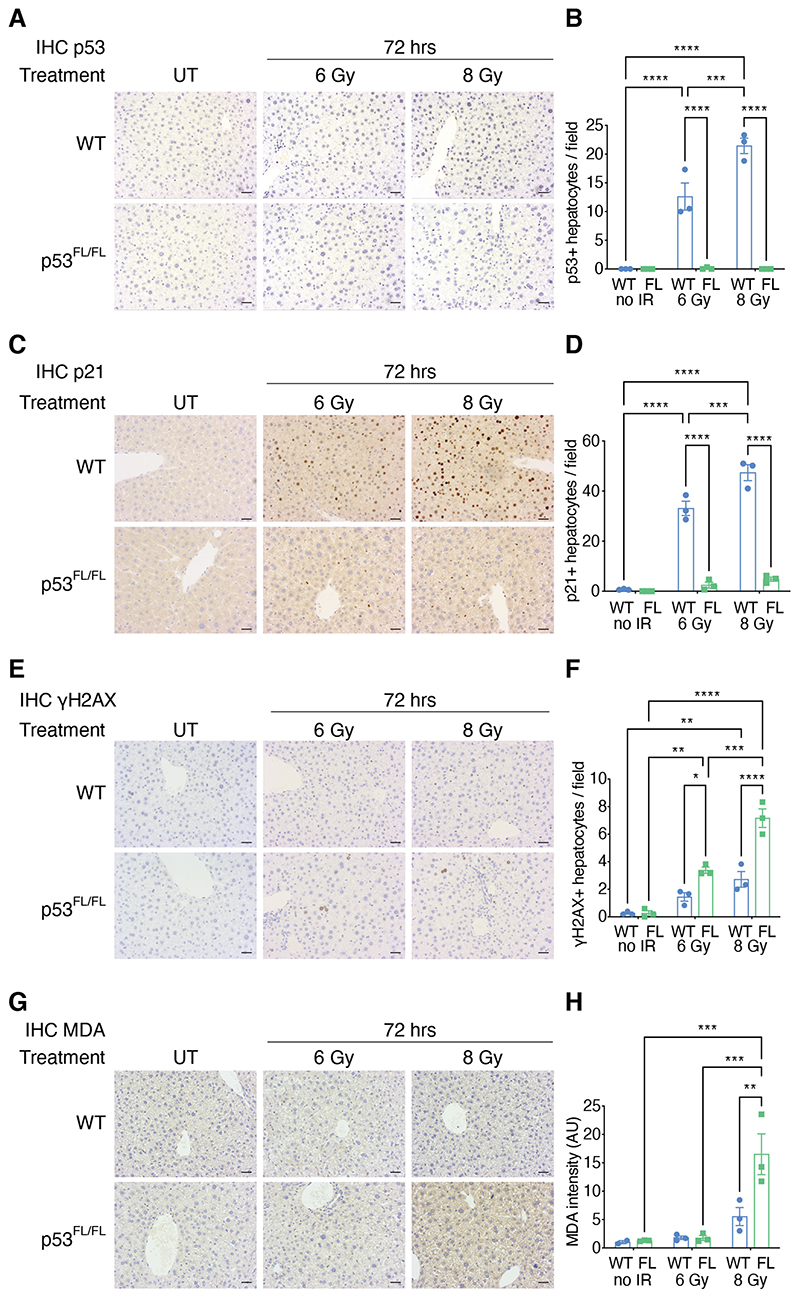
Liver p53 protects against DNA damage and ROS to maintain liver function after irradiation. **(A to H)** Representative IHC staining and quantification for (A and B) p53, (C and D) p21, (E and F) DNA damage by phospho-histone H2AX (γH2AX), and (G and H) ROS by malondialdehyde (MDA) abundance in hepatocytes from *Albumin*-Cre; *p53^WT/WT^* (WT) and *Albumin*-Cre; *p53^FL/FL^* (p53^FL/FL^ or FL) mice. N=3 mice per group, except for (G and H), wherein N=2 mice in the WT untreated group. Each data point represents the mean positives per field from 5 independent fields per mouse, presented alongside mean +/- SEM. *P<0.05, **P<0.01, ***P<0.001, and ****P<0.0001 by two-way ANOVA with Holm-Sidak’s multiple comparisons test and multiplicity-adjusted *P*-values. All scale bars, 20μm.

To explore the functional relevance of liver p53 in the response to TBI in adult mice, we generated *Albumin*-Cre; *p53^FL/FL^* mice (*p53^FL/FL^*) to induce liver-specific deletion of p53 during embryogenesis. Untreated *Albumin*-Cre; *p53^WT/WT^* (p53 WT) and *Albumin*-Cre; *p53^FL/FL^* mice were indistinguishable and exhibited similar liver histology (fig. S3E). At 3 days after TBI, we observed continued modest p53 stabilisation in *Albumin*-Cre; *p53^WT/WT^* hepatocytes that was greater in mice treated with 8 Gy TBI (Fig. 3, A and B), consistent with dose-dependent activation of p53 in the liver of these mice. As in our PG13-iRFP mice, we noted potent engagement of p21 throughout the liver in *Albumin*-Cre; *p53^WT/WT^* mice that also tracked with TBI intensity (Fig. 3, C and D). As expected, neither p53 nor p21 were detected in *Albumin*-Cre; *p53^FL/FL^* hepatocytes after either dose of IR, although we did observe increased p21 staining of immune cells in *Albumin*-Cre; *p53^FL/FL^* livers after TBI treatment (Fig. 3, A to D).

Ionizing radiation causes DNA damage and increased cellular reactive oxygen species (ROS) ^41^, and p53 has been shown to contribute to both DNA repair and ROS control ^42,43^. In *Albumin*-Cre; *p53^FL/FL^* mice we observed a substantial increase in unresolved DNA damage in the liver, as measured by phosphorylated histone H2AX (γH2AX) staining, in response to both sub-lethal and lethal TBI (Fig. 3, E and F). Furthermore, the *Albumin*-Cre; *p53^FL/FL^* livers exhibited a prominent increase in cellular ROS, as measured by malondialdehyde (MDA) staining, after 8 Gy TBI that did not occur in *Albumin*-Cre; *p53^WT/WT^* mice (Fig. 3, G and H). These findings suggest that induction of hepatocyte p53 signalling is important to resolve DNA damage and ROS arising from TBI.

### PG13-iRFP reporter mouse tracks acute activation of p53 during paracetamol and CCl_4_-mediated liver regeneration

**Figure 4:**
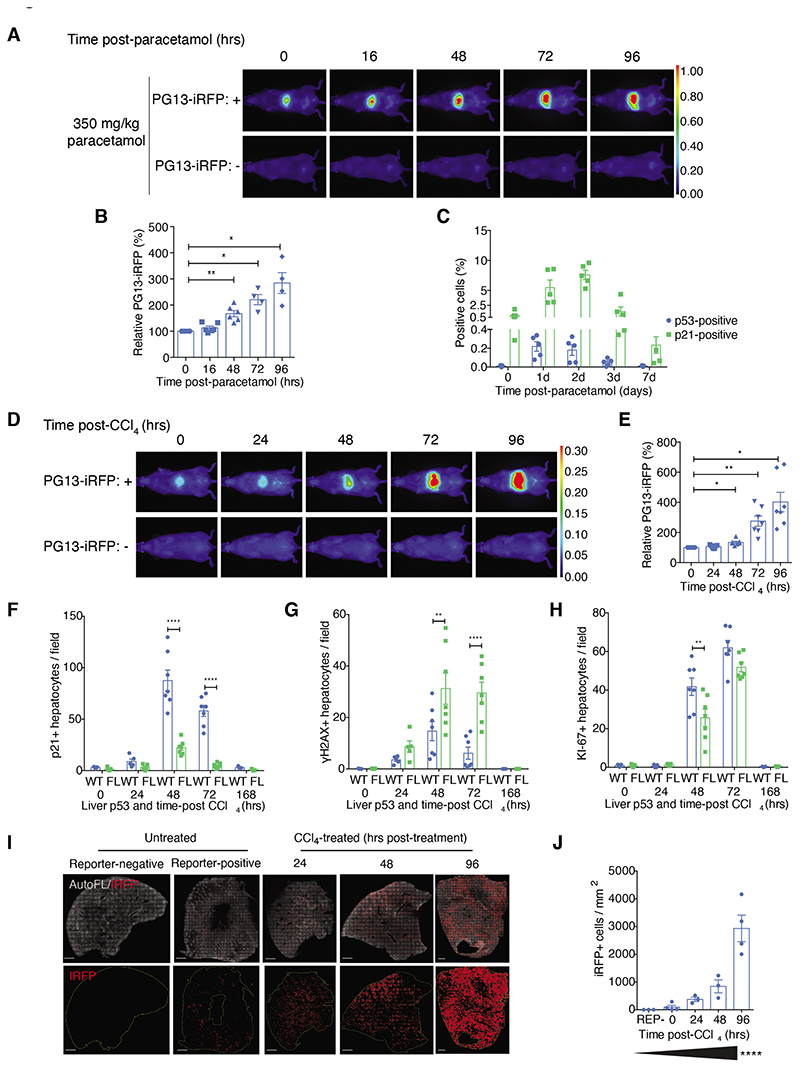
The PG13-iRFP reporter tracks potent liver-specific p53 activation during paracetamol and CCl_4_-mediated liver regeneration. **(A and B)** Representative images (A) of whole-body scans of liver-region iRFP intensity in PG13-iRFP+ and control PG13-iRFP-negative male mice at indicated time points (hours) after paracetamol treatment. Signal was quantified relative to baseline (time 0). Data (B) are mean ± SEM from N=2 PG13-iRFP-negative mice per time point; N=5 PG13-iRFP+ mice at time 0 and 16 and 48 hours after treatment; and N=4 mice at 72 and 96 hours after treatment. *P<0.05 and **P<0.01 using mixed-effects analysis with Holm-Sidak’s multiple comparisons test and multiplicity-adjusted *P*-values. (C) Analysis of IHC staining for p21 and p53 (shown as percentage of positive cells) from WT male mice at the indicated day (d) after paracetamol treatment. Data are mean ± SEM of N=5 mice per time point. **(D and E)** Representative whole-body scans (D) and quantification (E) of liver-region iRFP intensity relative to baseline (time 0) in PG13-iRFP+ and control PG13-iRFP-negative male mice after CCl_4_ treatment. N=7 PG13-iRFP+ mice per time-point. N=5 PG13-iRFP-negative mice at 0 and 48 hours, N=3 for 24 hours, and N=4 at 72 and 96 hours. Data are mean ± SEM; *P<0.05 and **P<0.01 using row-matched one-way ANOVA with Holm-Sidak’s multiple comparisons test and multiplicity-adjusted *P*-values. **(F to H)** Quantification of IHC staining for p21 (F), phospho- H2AX (gH2AX) (G), and Ki-67 (H) (positive hepatocytes per field) from *Albumin*-Cre; *p53^WT/WT^* (WT) and *Albumin*-Cre; *p53^FL/FL^* (FL) mice that were either untreated or analyzed after treatment with CCl_4_ at indicated time points (hours). Data are mean ± SEM from N=5 untreated mice WT and FL (at time 0), N=5 treated mice each at 24 hours, N=7 mice each at 48 and 72 hours, and N=3 mice each at 168 hours. **P<0.01 and ****P<0.0001 by two-way ANOVA with Holm-Sidak’s multiple comparisons test and multiplicity-adjusted *P*-values. **(I)** Representative images depicting auto-fluorescence (AutoFL, in white) and IRFP (in red) from Ce3D-cleared liver samples of untreated PG13-iRFP+ (Reporter-positive) and PG13-iRFP- (Reporter-negative) male mice and CCl_4_-treated PG13-iRFP+ male mice. N=3 mice each in the reporter-negative and 24- and 48-hour groups, and N=4 mice each in the PG13-iRFP+ untreated and 96-hour groups. Scale bar, 1mm. **(J)** Quantification of IRFP+ cells/mm^2^ from imaging represented in (I). Data are mean ± SEM; ****P<0.0001 by one-way ANOVA post-test for linear trend.

To assess the utility of the PG13-iRFP mice in detecting tissue-specific damage, we treated mice with paracetamol (acetaminophen), a commonly used analgesic/antipyretic but which at higher doses is hepatotoxic, following the established paracetamol-induced acute liver injury model^44–46^. In this system, p53 activity has been shown to play a significant role in protecting liver function and promoting liver regeneration^44–46^. Consistent with these reports, we observed increasing liver-region activation of the PG13-iRFP reporter over the first four days in male PG13-iRFP+ mice after 350mg/kg paracetamol treatment (Fig. 4, A and B). PG13-iRFP reporter activation coincided with increased p21 staining by immunohistochemistry (IHC) (Fig. 4C and fig. S4A), confirming that reporter signal reflected p53 activity during the response to paracetamol. However, although we noted broad activation of p21, this was not matched by similarly expansive stabilisation of liver p53 (Fig. 4C and fig. S4A), suggesting that activation of the p53 response to paracetamol treatment occurred without strong p53 stabilisation in the liver.

Expanding on these findings, we next examined whether p53 was similarly engaged during liver regeneration mediated by carbon tetrachloride (CCl_4_), another potent hepatotoxin ^47^. In the CCl_4_ acute regeneration model, extensive hepatocyte damage and cell death are followed by rapid proliferation and liver regeneration, allowing mice to survive this treatment^48^. As in the paracetamol model, male PG13-iRFP+ mice treated with CCl_4_ exhibited rising induction of iRFP in the liver from 2 days after treatment, peaking at 4 to 6 days after treatment, and returning to baseline by 14 days after treatment (Fig. 4, D and E, and fig. S4, B to D). The liver-specificity of the iRFP induction was confirmed in ex vivo tissues (fig. S4D).

Consistent with the observed PG13-iRFP induction, IHC analysis confirmed p53-dependent induction of p21 in *Albumin*-Cre; *p53^WT/WT^* mice in response to CCl_4_ that peaked at 2 days after treatment and returned to baseline within 7 days (Fig. 4F and fig. S4E). As in the paracetamol model, however, very little accumulation of p53 was detected by IHC in *Albumin*-Cre; *p53^WT/WT^* mice (fig. S4, F and G). Even so, loss of liver p53 exacerbated the intensity and duration of DNA damage after CCl_4_ treatment as judged by persistent IHC staining in the liver for phospho-H2AX in *Albumin*-Cre; *p53^FL/FL^* mice compared to *Albumin*-Cre; *p53^WT/WT^* mice (Fig. 4G and fig. S4H). Maximal proliferation, as judged by IHC staining for the proliferative marker Ki-67, was also delayed in *Albumin*-Cre; *p53^FL/FL^* mice compared to *Albumin*-Cre; *p53^WT/WT^* mice (Fig. 4H and fig. S4I). However, both of these deficits normalised by 7 days after CCl_4_ treatment, suggesting a transient protective role for p53 during CCl_4_-mediated regeneration, similar to that described in the paracetamol model^44,45^.

Although PG13-iRFP reporter signal was modestly increased by 2 days after CCl_4_ treatment, we noted an apparent disconnect between the low reporter intensity and extensive induction of p21 as assessed by IHC observed at this time point (Fig. 4, E and F). It is known that the extensive liver damage and cellular destruction arising from CCl_4_ treatment propagates radially outward from the central venous regions of the liver where CCl_4_ is initially activated by cytochrome P450 family protein CYP2E1^49,50^. We hypothesized that initial activation of p53 in this smaller subset of hepatocytes might not be resolvable on whole-body scans for iRFP. To address this possibility, and also to define the propagation of p53 activity throughout the liver during CCl_4_-mediated regeneration, we directly imaged iRFP in Ce3D-cleared liver sections^51^ from untreated PG13-iRFP-negative and positive reporter mice, as well as from PG13-iRFP+ mice at 24, 48, and 96 hours after CCl_4_ treatment (Fig. 4, I and J). In these cleared liver sections, we observed rising iRFP positivity after CCl_4_ treatment by 2 days (Fig. 4, I and J), consistent with p21 expression. In support of our model, the observed increase in iRFP-positive cells over time was well described by a quadratic equation (Y=B0 + B1*X + B2*X^2^) with an R^2^ value of 0.8582 (fig. S4J). Although this trend could be observed by examining the whole liver (Fig. 4, I and J), it was even more apparent in higher-magnification images focused on regions of liver damage (fig. S4K). When considered alongside the established protective role for p53 in the paracetamol acute liver damage model^44,45^, our findings are consistent with a similar paradigm of protective p53 activation following CCl_4_-mediated liver damage.

## Discussion

We have generated a novel iRFP p53 reporter that allows for rapid and non-invasive longitudinal visualisation of the p53 response in cell culture and in mice without requiring additional treatment with any substrates or supplements such as luciferin or biliverdin at each imaging session ^15–17,52^. Our cell culture studies indicate that activation of p53 by non-genotoxic signals (MDM2 inhibition by Nutlin or ribosomal stress by low level Actinomycin D) is more easily reversible than induction of p53 by DNA damaging agents, where the signal persists when the damaging signal is removed, likely reflecting the time taken to repair damage. Development of a PG13-iRFP p53 reporter mouse enabled us to identify increased p53 activity specifically in the brain, spine, and bones of E13.5 and E16.5 embryos. Interestingly, p53-null mice are susceptible to anencephaly, suggesting a role for p53 in supporting neural cell closure during development ^53^.

In adult mice, the iRFP reporter revealed low levels of p53 activity—notably in the liver—that was strongly induced in response to both sub-lethal and lethal total body irradiation. In agreement with published research, we observed rapid induction of iRFP signal in the gut-region of the PG13-iRFP mouse on whole-body scans that was matched to these organs *ex vivo*. However, this analysis also uncovered an unexpectedly strong induction of p53 activity in the radio-resistant liver and pancreas, findings that were validated through immunohistochemical staining for p21 in these tissues. Analysis of mice with liver-specific p53 deletion showed that this p53 response is important for protecting the liver during the acute response to TBI and allowing rapid repair of IR-induced damage. Activation of p53 in the liver was also seen in response to both paracetamol and carbon tetrachloride (CCl_4_) treatment. Compared to TBI, we observed a delayed and more time-limited increase in liver iRFP signal in response to either liver damaging treatment. These observations are consistent with an acute but rapidly resolved p53 response during acute liver damage and subsequent regeneration.

One caveat of our experimental system is that the long half-life of iRFP^16^ limits the ability of the model to monitor cessation of p53 activity in vivo. As such, it cannot be used to capture dynamic oscillations in p53 activation. However, because the stability of iRFP signal effectively marks tissues where p53 has been engaged, it provides an expanded period of time to identify these compartments after transient p53 activation for further analysis. In this way, the PG13-iRFP reporter mouse is a useful experimental model that allows comparative assessment of the induction of a p53 response over time following different stresses and in different organs non-invasively in a live mouse.

Our work introduces a new tool to study the dynamics of p53 activity both in vitro and in vivo, opening up avenues for research into the timing and spatial localisation of both canonical tumor suppressive and emerging non-tumor roles of p53 activity in the mouse. Using the PG13-iRFP model, we revealed a strong activation of the p53 response in the liver following IR, paracetamol, or CCl_4_ treatment. Previous studies have suggested a narrow and tightly controlled role for the p53 response in the liver, in part due to modest stabilization of liver p53 and reduced binding affinity for some target genes during acute hepatic stress ^54–57^. Although we also showed a very mild accumulation of p53 after either paracetamol or CCl_4_ treatment, our reporter mouse clearly revealed induction of p53-dependent signalling in both cases that promotes resolution of DNA damage. Further applications for the PG13-iRFP reporter mouse could be to gauge p53 activity during tumorigenesis, offering insights into optimal times to attempt therapeutic intervention in tumors that retain WT p53, for example. The PG13-iRFP mouse could also allow for broad classification of the induction of the p53 response in normal tissues in response to a variety of cancer therapies to help gauge potential on-target toxicities of new treatments. More broadly, the PG13-iRFP reporter mouse could be used as an exploratory platform to identify the timing and localisation of the p53 response to a variety of non-oncogenic stimuli such as during dietary over-feeding or restriction and for any p53 response in the development of non-cancer diseases.

## Materials and Methods

### Plasmids

The plasmid encoding P53rep-iRFP-destabilised (for in vitro expression) was generated by Gibson assembly cloning ^58^ using geneblocks (IDT). Briefly, full-length iRFP, created as previously described ^18^, was fused to a destabilisation domain to promote rapid degradation ^22^ (iRFP-deg). During the course of cloning, a mutation was inadvertently introduced into the iRFP protein (K99R) but this did not alter fluorescence characteristics of the resulting iRFP-deg construct. The iRFP-deg sequence was assembled together with a minimal CMV promoter linked with 5 repeats of consensus p53 response elements (P53 RE5) to yield a modified P53 RE5-minimal CMV (mCMV)-iRFP-deg-T2A-Luciferase reporter sequence modelled on the pGreenFire pathway reporter platform (System Biosciences). The specific P53 consensus elements used to create P53-RE_5_ were: 5’-GGACATGCCCGGGCATGTCC-3’ x 1, 5’-AGACATGTCCAGACATGTCC-3’ x 2, and 5’-GAACATGTCCCAACATGTTG-3’ x 2 and have been previously reported^19–21^.

For in vitro expression, the resulting construct was further cloned into a pEGFP-C1 plasmid backbone, modified during PCR amplification to remove the existing CMV promoter (Clontech #6084-1) and used to generate stable P53rep-iRFP-expressing U2OS cells as described below.

The plasmid encoding PG13-iRFP (used to make the in vivo targeting construct) was modelled on the PG13 p53 response element construct ^23^ and generated by Gibson assembly cloning ^58^ using geneblocks (IDT) and PCR fragments. The PG13 sequence and polyoma virus early gene promoter (Py) were PCR amplified from the PG13-luc plasmid (Addgene #16442)^23,29^. Full-length iRFP was fused downstream of the Py promoter, replacing the luciferase of the original PG13-luc plasmid. These features comprised the core PG13-Py-iRFP domain. As with the in vitro P53 RE_5_-mCMV-iRFP-deg-T2A-Luciferase reporter sequence, the PG13-Py-iRFP domain was further cloned into the modified pEGFP-C1 plasmid backbone described above.

### Generation of PG13-iRFP knock-in mice

The PG13-iRFP construct, as described above, was PCR amplified and cloned into the plasmid pSKB1^59^ following sequence validation. The pSKB1 plasmid comprises 5’ and 3’ homology arms for targeting to the deleted *Hprt* locus of HM1 embryonic stem cells, simultaneously regenerating a functional *Hprt* gene (allowing selection under hypoxanthine-aminopterin-thymidine (HAT) medium).

8×10^6^ mid-log phase HM1 embryonic stem cells ^60^ were re-suspended in Embryomax electroporation solution (Millipore cat # ES-003-D) and mixed with 40μg of AscI-linearised targeting vector. Electroporation was performed under standard conditions (250V; 500μF; infinite resistance; cuvette width: 4mm) in a Bio-Rad GenePulser XCell with capacitance extender. Under these conditions, the time constant is close to 7 msecs. After plating onto DR4 irradiated mouse embryonic fibroblast (MEF) monolayers ^61^ across four 10cm plates, cells were maintained under regular ESC medium for 40-48 hours before being placed under hypoxanthine-aminopterin-thymidine (HAT) selection for 5 days.

Correct targeting of the vector to the *Hprt* locus on both the 5’ and 3’ sides was confirmed using PCR on genomic DNA prepared from HAT-resistant colonies. PCR genotyping was done using Expand Long Template (Sigma Aldrich cat # 11681834001) according to the manufacturer’s recommendations. Oligo sequences used to screen cells to ensure appropriate targeting of the *Hprt* gene were: GTTGCTGAGGCAAAAATAGTGTAAT and GAATGCAATTGTTGTTGTTAACTTG for the 5-prime side and: CTACCTAGTGAGCCTGCAAACTG and ATGTAAGTGCTAGGAATTGAACCTG for the 3-prime side.

Following identification of correctly targeted clones, mouse lines were derived by injection of ES cells into C57BL/6J blastocysts according to standard protocols ^62^. After breeding of chimeras, germline offspring were identified by coat colour and the presence of the modified allele was confirmed by PCR specific for the iRFP transgene, using the primers: ACAACCTTCCCGAACTCACC and CGATCCTCTGCGATCACTTC.

### Mice

Procedures involving mice were performed under Home Office licence numbers 70/8645, 70/8468, and 70/8891. Experiments were conducted in accordance with the Animals (Scientific Procedures) Act 1986 and the EU Directive 2010 and sanctioned by Local Ethical Review Process (University of Glasgow). Mice were housed on a 12-hour light/12-hour dark cycle in groups of 3 to 5 as much as possible. Mice were provided with environmental enrichment in the form of Sizzle-Nest bedding and polycarbonate tunnels, normal chow diet, and water ad libitum. Mice were genotyped by Transnetyx (Cordova, TN). *p53* ^FL/FL^ (Trp53^tm1Brn^), *Albumin*-Cre (Speer6-ps1^Tg(Alb-cre)21Mgn^), and *p53^KO/KO^ (Trp53^tm1Brd^)* mice were described previously ^63–65^ and were fully backcrossed to C57BL6/J (N10).

For all imaging experiments, mice were shifted onto a specially formulated low-fibre ‘IRFP’ diet (AIN-93M Purified Diet, Envigo) for at least 7 days in order to eliminate non-specific fluorescence in the gut prior to pre-treatment baseline imaging. Mice continued to be maintained on IRFP diet for the duration of each experiment. For the imaging of embryos, pregnant mice were shifted onto IRFP diet 7 days prior to imaging.

For total body irradiation (TBI) experiments, mice were treated as previously described^33^ with either 6 or 8 Gy TBI from an Xstrahl RS225 Cabinet X-ray Irradiator (Xstrahl) as indicated, imaged at the time points indicated, and sampled as noted in the manuscript.

For the paracetamol acute liver injury model, mice were treated as previously described^44–46^. In brief, young male C57BL/6J mice (approx. 70 days old) were fasted for a period of 9 hours (food taken away at 07:00 and put back at 16:00) and then given paracetamol at a dose of 350mg/kg. To prepare the paracetamol stock solution, 350mg of paracetamol powder (Sigma Aldrich, A7085) was dissolved in 20mL sterile PBS. Mice were injected with 20μl of the stock paracetamol solution per gram body weight by a single intraperitoneal (i.p.) injection administered at 4pm in the afternoon.

For CCl_4_-mediated liver regeneration, mice were treated as previously described ^66,67^. In brief, young male C57BL/6J mice (approx. 70 days old) were given CCl_4_ (1mL/kg from stock solution of 20% CCl_4_ v/v in corn oil) (Sigma cat #289116 and #C8267) by a single i.p. injection administered in the morning.

The 6 Gy TBI 50-day post treatment and control mice were female. Otherwise, irradiation cohorts were a mix of male and female mice. Liver regeneration mice were all male for both paracetamol and CCl_4_ experiments. Mice within each experiment were age and littermate matched as much as possible, and all were started on experimental procedures and/or imaging prior to 6 months of age. Downstream analyses were performed on a random order of samples blinded to the genotype and treatment regime of a given sample until the summation of results.

### In vivo imaging

Mice were imaged on a Pearl Impulse Small Animal Imaging System (LI-COR) as previously described^15^. LI-COR supplied filter sets and light sources were used for all imaging sessions and iRFP emission (685nm) was detected in the 700nm channel. No exogenous biliverdin was introduced in any of these experiments. Scan images are presented with false colour LUTs. Imaging parameters were kept constant across time points and mice within an experiment. For quantification, Image Studio software (LI-COR, V5.2) was used.

For experiments involving embryos, pregnant mice were euthanized by schedule 1 method prior to analysis. Mice were scanned on the Pearl Imaging System to obtain the whole-body scans presented in Figure 1H. Embryos were subsequently removed from the embryo sac and scanned at higher resolution directly on an Odyssey LI-COR scanner (Li-COR) in Figure 1I. iRFP emission (685nm) was detected in the 700 channel with embryos scanned at 42μm resolution on “high” quality with a 4.0mm offset and a low-intensity setting (1.0) held constant throughout each experiment.

### Measuring p53 activity in vitro

The measuring of in vitro p53 activity through iRFP expression was performed as described previously for monitoring cell growth via iRFP ^18^. Briefly, stable U2OS cell lines expressing the in vitro P53rep-iRFP construct were seeded at a density of 3000 cells per well in 96-well CellBIND black microplates with clear flat bottom (Corning cat # 3340) and allowed to settle. The next day, medium was changed, and cells were treated with compounds or vehicle control as indicated in the manuscript. Treatment continued for a period of ~40 hours before compounds were washed out via complete replacement of the medium in each well. Throughout each experiment, iRFP intensity was measured using an Odyssey Li-Cor scanner. iRFP emission (685nm) was detected in the 700 channel. For quantification, plates were scanned at 169μm resolution with a 3.5mm offset and a low-intensity setting held constant throughout each experiment. Image Studio software (LI-COR, V5.2) was used to scan and subsequently quantify the plates.

### Immunohistochemistry (IHC)

All IHC staining was performed on 4μm formalin fixed paraffin embedded (FFPE) sections that had previously been warmed at 60°C for 2 hours. Slides were de-paraffinised and rehydrated according to the relevant staining platform for each antibody as noted in the accompanying reagent and antibody information tables (tables S1 and S2). Manual staining for MDA was performed as described previously ^68^ using the reagents recorded in the information table.

For staining on the Dako Autostainer Link 48 (Agilent, UK), FFPE sections underwent dewaxing and heat induced epitope retrieval (HIER) on-board a Dako PT module. Once loaded, the sections were heated to 97°C for 20 minutes using Target Retrieval Solution (TRS), high pH (Agilent, UK). The sections underwent peroxidase blocking (Agilent, UK) before application of primary antibody for 40 minutes at a previously optimised dilution. Secondary antibody incubation and downstream signal detection were performed using the rabbit envision secondary antibody for 30 minutes and Liquid DAB (both Agilent, UK).

For staining on the Leica Bond Rx autostainer, sections were loaded onto the autostainer and underwent dewaxing and epitope retrieval on board using ER2 buffer for 30 minutes at 95°C (Leica, UK). Sections were rinsed with Leica wash buffer before undergoing peroxidase block for 5 minutes using an Intense R kit (Leica, UK). Sections were incubated with a previously optimized concentration of primary antibody for 30 minutes. Secondary antibody incubation was performed using the indicated secondary antibody kit for 30 minutes and signal was visualized with DAB using the Intense R kit (Leica). After detection in either system or manually, sections were counterstained with haematoxylin and coverslipped using DPX mountant (CellPath, UK) prior to analysis.

### Analysis of IHC images

For quantification of p53, p21, KI-67, and phospho-H2AX in irradiation experiments, five random non-overlapping 20X magnification images were taken from each IHC slide using an Olympus BX51 microscope with Zen Blue software (Zeiss). From these images, positive cells per 20X field were quantified by hand and the mean value per mouse was reported in the relevant figures. Quantification of Malondialdehyde (MDA) staining intensity was performed as previously described ^68^, from images acquired as described above. All samples for each IHC stain for a given experiment were quantified at the same time, blinded to the genotype and treatment until final tabulation of the counts.

The quantification of p53 and p21 positive hepatocytes in paracetamol experiments was performed using the HALO imaging and analysis platform (v3.1.1076.363, Indica Labs). Briefly, after annotating the desired tissue area (3-4 liver lobes) an algorithm was built to detect positively stained nuclei, based on the “Indica Labs - CytoNuclear v2.0.9” built-in template. All samples were run in the same batch and with the same algorithm.

### Fluorescence imaging of cleared liver samples

Harvested livers were fixed overnight in formaldehyde (Thermo Scientific 4% v/v in PBS). Livers were then cut with a vibrating microtome (Campden 5100mz, Campden Instruments LTD, UK) into 250 μm slices. Samples were permeabilized for 1h in PBS/Normal goat serum (NGS; Merck) 10%/BSA1%/TritonX-100 (TX-100) 0.3%/Azide 0.05% and stained with DAPI (Thermo Scientific; 1:1000) for 1h. Samples were then washed 3 times for 10 minutes in PBS/BSA1%/TX-100 0.1%/Azide 0.05%, 2 times in PBS and subsequently fixed in 4% formaldehyde. Samples were then cleared for 45 minutes using Ce3D clearing solution prepared as described^51^ and slices were transferred to glass slides, covered with Ce3D and coverslipped.

Tiled images were acquired with a Zeiss LSM 880 NLO multiphoton microscope (Carl Zeiss, Germany) equipped with a 32-channel Gallium Arsenide Phosphide (GaAsP) spectral detector (Carl Zeiss, Germany) using 20×/1 NA water immersion objective lens as previously described^69^. Samples were either excited with a tunable pulsed laser in multiphoton mode (Discovery, Coherent, USA) set at 1000 nm, or with the combination of 405, 488 and 633 continuous wave lasers for single-photon operation. DAPI, autofluorescence and second harmonic generation signals were collected onto the GaAsp array in lambda mode with a resolution of 8.9 nm over the visible spectrum (410-696nm). iRFP signals were collected using a Photo Multiplier Tube (PMT) detector (697-750nm). Spectral images were then unmixed with Zen Black software (Carl Zeiss, Germany) using reference spectra from unstained tissues (tissue autofluorescence, second harmonic generation and iRFP) or DAPI-stained tissue. When necessary, 2-photon and single photon images were merged and stitched using Zen Blue (Carl Zeiss, Germany).

Fluorescence images were analysed with QuPath^70^. Full liver sections were annotated using the “simple tissue detection” tool. P53 activity was estimated using the “positive cell detection” tool and expressed as number of iRFP+ cells/mm2 of tissue.

### Cell culture

U-2 OS (U2OS) human osteosarcoma cells were obtained from ATCC (cat # HTB-96). Stock flasks of cells were maintained in DMEM high glucose medium containing pyruvate (Gibco, 21969035) supplemented with 2 mM L-glutamine, penicillin/streptomycin, and 10% FBS. Cells were cultured at 37°C in a humidified atmosphere of 5% CO_2_. Mycoplasma testing was performed semi-regularly and cells were negative throughout in vitro experiments.

Stable U2OS cell lines expressing the in vitro P53rep-iRFP construct were created by transfecting parental U2OS cells with the P53rep-iRFP plasmid using Genejuice (Sigma Aldrich cat # 70967-3) according to the manufacturer’s instructions, followed by selection with 500μg/ml G-418 (Sigma Aldrich cat # 4727878001). Drug-resistant cells were then re-seeded sparsely, and clonal colonies were picked.

U2OS cells were treated with the following compounds: Nutlin-3A (1, 2, 5, or 10 μM in DMSO; Sigma cat # N6287), actinomycin D (2.5 or 5 nM in DMSO; UCL Pharmacy), 5-fluorouracil (5 or 10μg/mL in DMSO; Sigma cat # F6627), adriamycin (doxorubicin; 100 or 200ng/mL dissolved in H2O; Sigma cat # 324380), etoposide (10 or 20 μM in DMSO; Sigma cat# E1383) or cisplatin (3.75 or 7.5 μg/mL in DMSO; Sigma cat #232120). DMSO or H_2_O were used as vehicle control treatments as appropriate. Experiments contained at least 4 technical replicates per condition and were repeated at least three times.

### Data plotting and statistical analysis

Data were plotted using Prism 7 (Graph Pad). The statistical analysis for each experiment was performed using the test indicated in the relevant figure legend and multiplicity-adjusted p-values using the built-in analysis tools of Prism 7. For most experiments, this analysis was either a one-way ANOVA or multiple t-tests, adjusted using Holm-Sidak’s protocol. Statistical analyses were independently reviewed prior to publication. Figures were prepared using Illustrator (Adobe). Unless otherwise indicated, data are represented as mean ± standard error of the mean (SEM). Asterisks denote p-value thresholds as indicated in the legends.

## Supplementary Material

Fig. S1

## Data Availability

All data needed to evaluate the conclusions in the paper are present in the paper or the Supplementary Materials.
