# Supplementary material for: A non-invasive iRFP713 p53 reporter reveals dynamic p53 activity in response to irradiation and liver regeneration in vivo†
Source: Sci Signal. Author manuscript; Available in PMC 2022 Mar 7. (PMC7612476; doi:10.1126/scisignal.abd9099)
Supplement: Fig. S1 [file EMS143776-supplement-Fig__S1.pdf]

Science Signaling
AAAS

Supplementary Materials for

A noninvasive iRFP713 p53 reporter reveals dynamic p53 activity in response to irradiation and liver regeneration in vivo

Timothy J. Humpton *et al.*

Corresponding author: Karen H. Vousden, karen.vousden@crick.ac.uk; Timothy J. Humpton, t.humpton@crick.ac.uk

Sci. Signal. **15**, eabd9099 (2022)
DOI: 10.1126/scisignal.abd9099

This PDF file includes:

Figs. S1 to S4
Tables S1 and S2

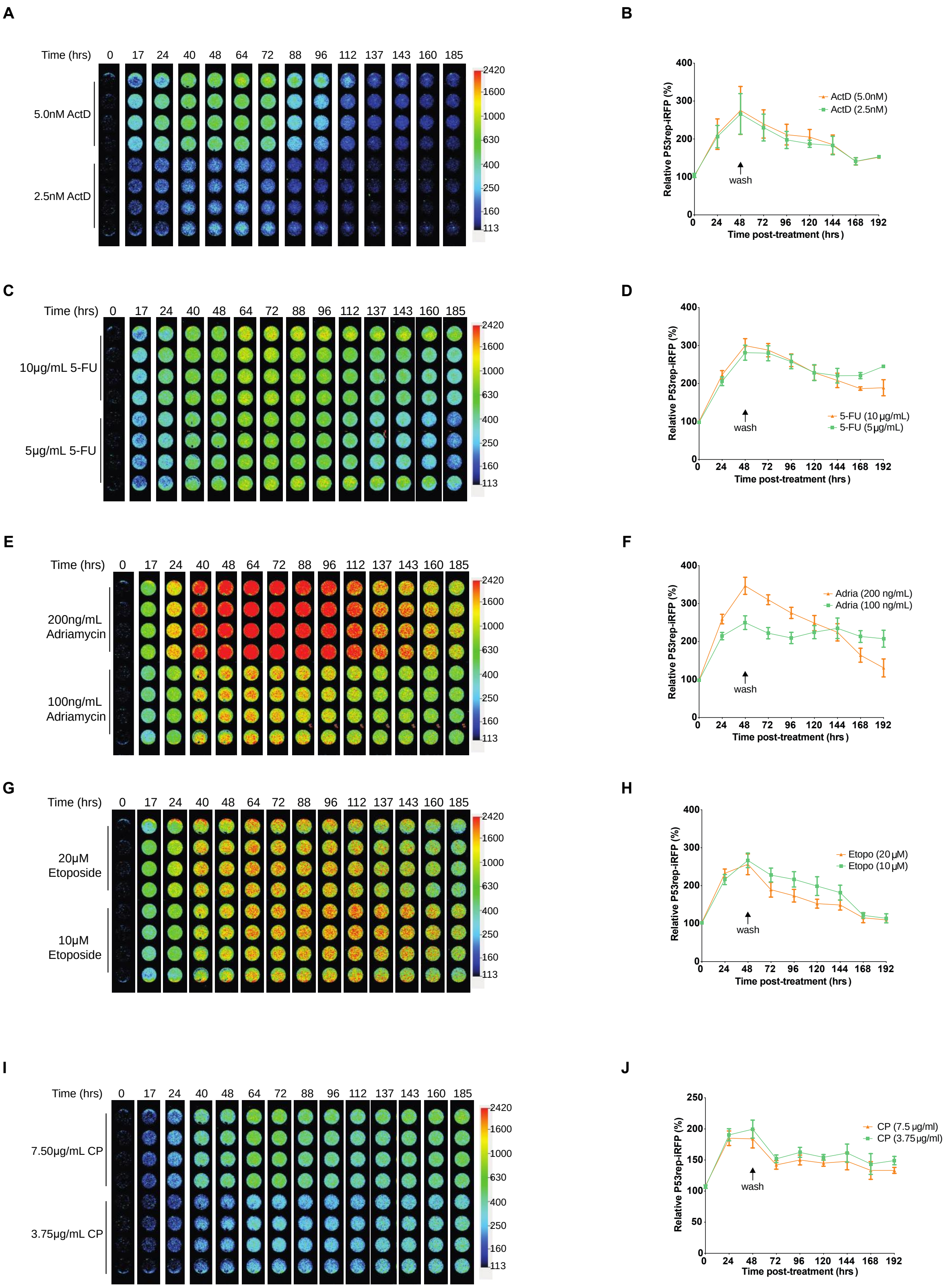

Figure S1: Validation of P53rep-iRFP reporter in vitro. (A to J) Time-series (hours post-treatment) and quantification of iRFP relative to matched untreated baseline iRFP level in stable P53rep-iRFP+ U2OS cells treated with (A and B) 5nM or 2.5nM Actinomycin D (ActD), (C and D) 10µg/mL or 5µg/mL 5-fluorouracil (5-FU), (E and F) 200ng/mL or 100ng/mL Adriamycin (Adria), (G and H) 20µM or 10µM Etoposide (Etopo), or (I and J) 3.75 or 7.5µg/mL Cisplatin (CP) with compounds removed at 48 hours ("wash"). Representative images are from technical replicates of one experiment and quantification is from N=6 independent experiments; data presented as mean ± SEM.

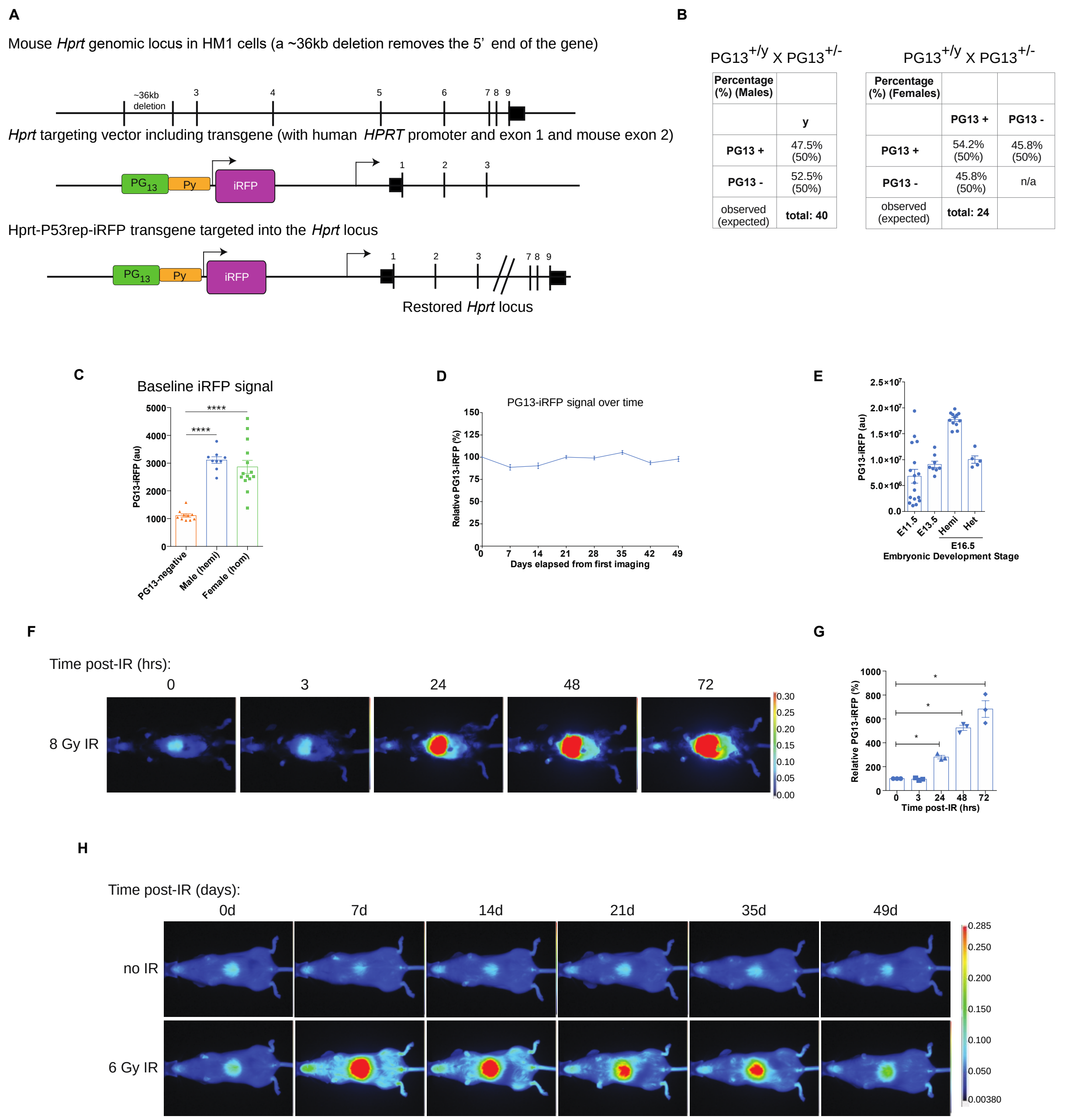

Figure S2: Validation of PG13-iRFP reporter in vivo. (A) Schematic of PG13-iRFP in vivo targeting strategy. Further details in methods. **(B)** Genotype distribution (%) amongst live births from mice arising from cross between PG13-iRFP hemizygous male ($PG13^{+/y}$) mice with PG13-iRFP heterozygous female ($PG13^{+/-}$) mice. Analysis of N=64 live births from 9 mating pairs. For female mice, no PG13 homozygous-negative females were expected or born from this cross (n/a) since the allele is X-linked. **(C)** Baseline iRFP signal (AU) in PG13-iRFP-negative and PG13-iRFP positive male (hemizygous) and female (homozygous) mice at first imaging session. N=9/14 male/female PG13-iRFP+ and N=4/6 male/female WT (PG13-negative) mice (pooled). Data presented as mean ± SEM and analysed using one-way ANOVA with Holm-Sidak's multiple comparisons test: ****P<0.0001. **(D)** Quantification of iRFP signal relative to initial imaging session of PG13-iRFP+ mice from (C) measured over time (days elapsed from first imaging session). Data from N=3 mice (2 female, 1 male) and graphed as mean ± SEM. **(E)** Quantification of images of ex vivo iRFP signal from embryos at days E11.5, E13.5, and E16.5 arising from timed matings between $PG13\text{-}iRFP^{+/+}$ female and $PG13\text{-}iRFP^{+/y}$ male mice (E11.5 and E13.5) or from $PG13\text{-}iRFP^{+/+}$ female and PG13-iRFP-negative (WT) male mice (E16.5). N=17 E11.5 embryos, N=8 E13.5 embryos, and N=17 E16.5 embryos from N=2 timed matings per time point. Data presented as mean ± SEM with individual embryos shown. E11.5 and E13.5 embryos are grouped together due to equivalent genotypes in all embryos (either hemizygous (male) or homozygous (female) for PG13-

iRFP). E16.5 embryos are presented by genotype for PG13-iRFP [hemizygous (Hemi) or heterozygous (Het)]. **(F and G)** Representative images and quantification of PG13-iRFP+ mice treated with 8 Gy TBI and imaged at 3 hours after irradiation and then as indicated. LUT profile used for all images shown. Representative of N=3 mice. The mice used in this experiment had black coat colour and all mice were depilated prior to baseline imaging. Data presented as mean +/- SEM and analysed using one-way ANOVA with Holm-Sidak's multiple comparisons test: $^*P<.05$. **(H)** Long-term images (days post-IR) of PG13-iRFP+ mice that were either untreated (no IR) or treated with 6 Gy TBI (N=2 no IR and N=3 IR) and monitored for PG13-iRFP signal over time. LUT profile used for all images shown. Mice shown are the same as those depicted in the acute IR time-course (UT and 6 Gy PG13-iRFP+) in Fig. 1I and therefore the time 0 images are the same in these figures.

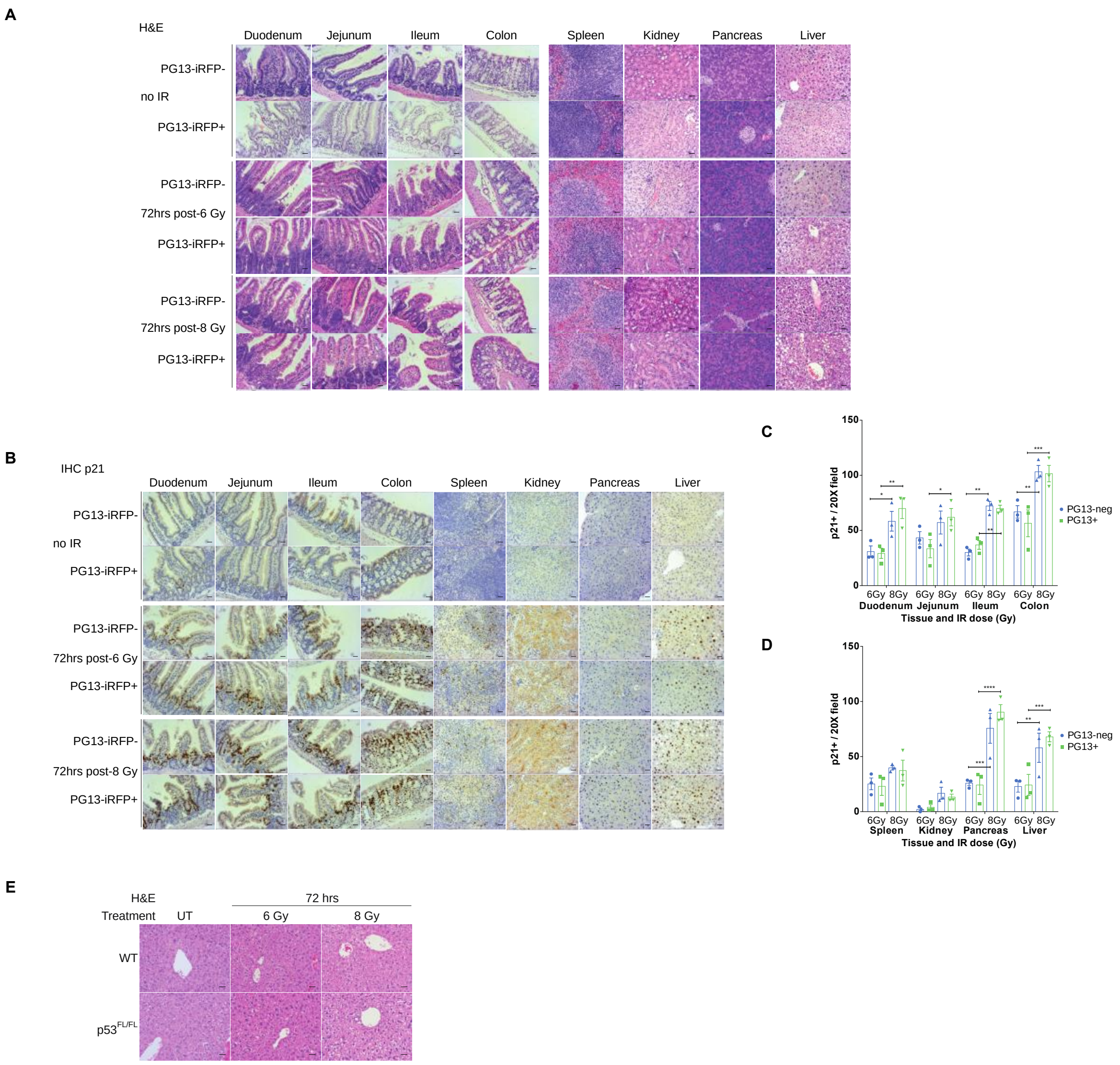

Figure S3: PG13-iRFP reporter mouse confirms differential p53 activity across tissues after IR. (A) Representative H&E staining in WT (PG13-iRFP-) and PG13-iRFP+ mice either untreated (no IR) or at 72 hours after 6 Gy or 8 Gy TBI treatment. N= 3 mice/group. Scale bars 20μm. **(B-D)** Representative IHC staining (B) and quantification (C and D) for p21 in WT (PG13-iRFP-) and PG13-iRFP+ mice from (A). N= 3 mice/group. Scale bars 20μm. Each data point represents the mean number of p21-positive nuclei per 20X field from 5 independent fields per mouse. Data presented as mean ± SEM and analysed using two-way ANOVA with Holm-Sidak's multiple comparisons test and multiplicity-adjusted *P*-values: **P*<0.05, ***P*<0.01, ****P*<0.001, ****p<.0001. **(E)** Representative H&E staining of livers from *Albumin*-Cre; $p53^{WT/WT}$ (WT) and *Albumin*-Cre; $p53^{FL/FL}$ ($p53^{FL/FL}$) mice that were either untreated (UT = no IR) or analysed at 72 hours after 6 Gy or 8 Gy TBI treatment. N=3 mice per group. Scale bars 20μm.

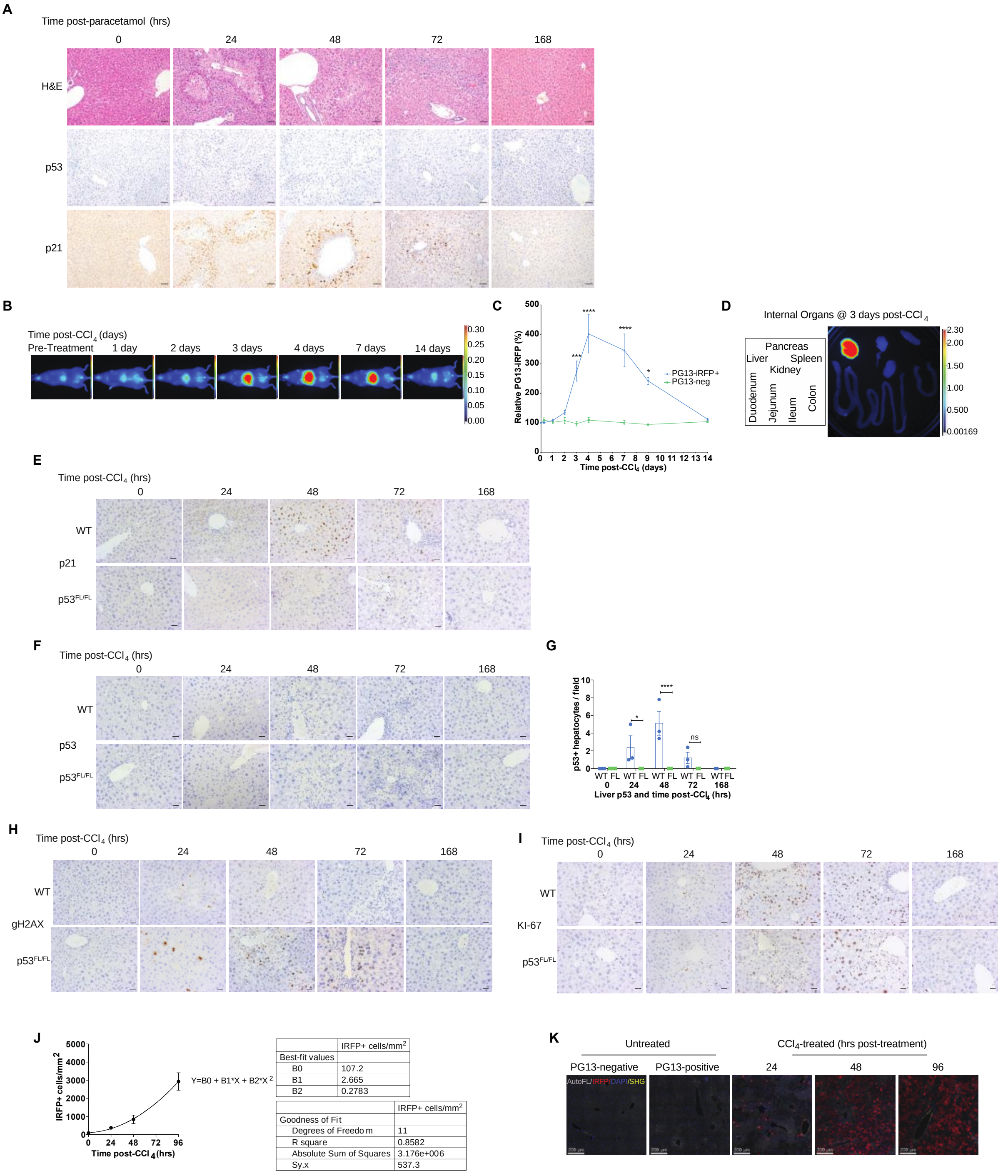

Figure S4: PG13-iRFP reporter mouse confirms p53 activity during paracetamol and CCl_4-mediated liver damage and regeneration. (A) Representative H&E and IHC staining for p21 and p53 in WT male mice at indicated time points (days) after paracetamol treatment. Representative of N=5 mice per time point. Scale bars 20µm. **(B and C)** Representative whole-body scans (B) and quantification (C) of liver-region iRFP intensity in PG13-iRFP+ male mice during CCl_4-mediated liver regeneration (hours per days after CCl_4 treatment as indicated). N=7 PG13-iRFP+ mice for pre-treatment and days 1 to 4, N=6 for 6 hours, N=5 for day 7, and N=3 for days 9 and 14. N=5 WT mice for pre-treatment, N=4 for 7hrs, N=3 for day 1, N=5 for day 2, N=4 WT mice for days 3 and 4, and N=3 for days 7, 9, and 14. LUT profile used for all images shown. Data are mean +/- SEM and analyzed using two-way ANOVA with Holm-Sidak's multiple comparisons test and multiplicity-adjusted *P*-values: **P*<0.05, ****P*<0.001, and *****P*<0.0001. **(D)** Ex vivo analysis of organs of PG13-iRFP+ mouse at 3 days post-CCl_4 treatment. LUT profile used for all ex vivo images as shown. Representative of N=3 mice. Layout of tissues within the images is as shown. Tissue location is not meant to reflect actual positioning within the abdominal cavity. **(E)** Representative IHC staining for p21 from *Albumin*-Cre; *p53*$^{WT/WT}$ (WT)

and *Albumin*-Cre; *p53*$^{FL/FL}$ (p53$^{FL/FL}$) mice that were either untreated (0 hours) or analysed after treatment with CCl_4 at the indicated time points (hours). N=5 untreated mice/group, N=5 mice per group at 24 hours, N=7 mice per group at 48 and 72 hours, and N=3 mice per group at 168 hours after treatment. Scale bars 20µm. Relates to quantifications shown in Fig. 4F. **(F and G)** Representative images (F) and quantification of IHC staining for p53 (G) (positive hepatocytes / field) from *Albumin*-Cre; *p53*$^{WT/WT}$ (WT) and *Albumin*-Cre; *p53*$^{FL/FL}$ (p53$^{FL/FL}$) mice that were either untreated (0 hrs) or analysed after treatment with CCl_4 at indicated time points (hrs). Representative of N=4 untreated mice/group and N=3 treated mice per group at each time point. Data are mean ± SEM and analysed using two-way ANOVA with Holm-Sidak's multiple comparisons test and multiplicity-adjusted p-values: **P*<0.05 and *****P*<0.0001. **(H and I)** Representative IHC staining for phospho-Histone H2AX (gH2AX) (H) and KI-67 (I) from *Albumin*-Cre; *p53*$^{WT/WT}$ (WT) and *Albumin*-Cre; *p53*$^{FL/FL}$ (p53$^{FL/FL}$) mice treated as in (F). Representative of N=5 untreated mice/group, N=5 mice/group at 24 hours, N=7 mice/group at 48 and 72 hours, and N=3 mice per group at 168 hours after treatment. Scale bars 20µm. Relates to quantifications shown in Fig. 4, G and H. **(J)** Curve-fit analysis of the quantification of IRFP+ cells/mm^2 from images in Fig. 4I. N=4 PG13-iRFP+ untreated mice, N=3 mice 24 and 48 hours after treatment, and N=4 mice 96 hours after treatment. Data presented as mean ± SEM and analysed for second-order polynomial (quadratic) curve-fit. Derived equation parameters and goodness-of-fit values for resulting curve as shown. **(K)** Representative fluorescent images depicting auto-fluorescence (AutoFL, in white) DAPI (in blue), and IRFP (in red), and second harmonic generation (SHG; in yellow) from Ce3D-cleared liver samples of PG13-iRFP-positive and PG13-iRFP-negative (REP-) untreated male mice and PG13-iRFP+ male mice treated with CCl_4 for the indicated times (hours). Representative of N=3 REP- and N=4 PG13-iRFP+ untreated mice, N=3 mice 24 and 48 hours after treatment, and N=4 mice 96 hours after treatment. Scale bars 200µm. Further details are in the Methods.

Reagent	Company	Code
ER2	Leica	AR9640
Liquid DAB	Agilent	K3486
Rabbit EnVision	Agilent	K4003
Rat ImmPRESS	Vector Labs	MP-7404
TRS High pH antigen retrieval solution	Agilent	K4008
Citrate-based antigen unmasking solution	Vector Labs	H-3300
BLOXALL blocking solution	Vector Labs	SP-6000
Vectastain ABC Elite kit	Vector Labs	PK-6010
ImmPACT DAB	Vector Labs	SK-4105
Bond Dewax Solution	Leica	AR9222

Table S1: IHC reagents

Antibody	Company	Code	Autostainer	Retrieval	Dilution
Phospho-H2A.X	CST	9718	Leica Bond Rx	ER2	1:120
p21	Abcam	ab107099	Leica Bond Rx	ER2	1:150
KI-67	Abcam	ab16667	Leica Bond Rx	ER2	1:1000
p53	Leica	NCL-L-p53-CM5p	Dako Link 48	TRS High pH	1:750
MDA	Abcam	ab6463	manual	Boiling Citrate	1:333

Table S2: Primary antibodies used for IHC
